# Effectiveness and safety of a mumps containing vaccine in preventing laboratory-confirmed mumps cases from 2002 to 2017: A meta-analysis

**DOI:** 10.1515/biol-2022-0820

**Published:** 2024-02-08

**Authors:** Bu-Gang Gao, Ling-feng Huang, Ping Xie

**Affiliations:** Rehabilitation Teaching and Research Office, Department of Medicine, ChuZhou City Vocational College, Chuzhou, Anhui Province, China; Department of Epidemiology and Biostatistics, School of Public Health, Guangdong Medical University, Zhanjiang, Guangdong, China; Community Health Service Center in Nantou, Zhongshan, Guangdong Province, China

**Keywords:** mumps containing vaccine, meta-analysis, disease prevention, viral diseases, acute respiratory infection

## Abstract

Emerging evidence has figured that serum conversion rate of mumps is a crucial link of mumps disease. Nevertheless, a rising number of mumps outbreaks caused our attention and studies examining the serum conversion cases were conducted in small samples previously; this meta-analysis was conducted to assess the immunogenicity and safety of a mumps containing vaccine (MuCV) before 2019. We identified a total of 17 studies from the year of 2002–2017. In the case–control studies, the vaccine effectiveness (VE) of MuCV in preventing laboratory-confirmed mumps was 68% (odds risk: 0.32; 95% confidence interval [CI], 0.14−0.70) while in the cohort studies and randomised control trials, 58% (relative risk [RR]: 0.42; 95% CI, 0.26−0.69). Similar intervals of effectiveness rates were found during non-outbreak periods compared with outbreak periods (VE: 66%; RR: 0.34; 95% CI, 0.18−0.68 versus VE: 49%; RR: 0.51; 95% CI, 0.21−1.27). In addition, the MuCV group with two and three doses did not show enhanced laboratory-confirmed mumps than one dose (VE: 58%; RR: 0.42; 95% CI, 0.20−0.88 versus VE: 65%, RR: 0.35; 95% CI, 0.20−0.61) for the reason of the overlap of 95% CI. MuCV had comparable effectiveness comparing non-outbreak and outbreak period, one dose, and two or three doses. MuCV displayed acceptable adverse event profiles.

## 1 Introduction

Mumps is an acute respiratory infection caused by mumps virus infection of the parotid gland [1]. Mumps is highly transmissible as a common childhood viral disease and is also prevalent in both teenagers and young adults. Mumps should be a global concern, especially in China due to the considerable number of mumps cases [2]. Previous studies have reported the highest incidences of mumps in China in 2011 and 2012, with the incidence 33.9/100,000 and 35.6/100,000 [2]. Mumps is a self-limited disease that is mild in most cases but has serious complications, including meningitis and orchitis [3]. Examination of laboratory-confirmed mumps is an important indicator of exposure to the mumps virus.

Mumps is a vaccine-preventable disease. Widespread use of mumps containing vaccine (MuCV) has dramatically decreased the incidence of mumps. Mumps population immunity depends on the widespread use of MuCV [1]. Immunization with a MuCV is recommended by the World Health Organization, and the widespread use of MuCV has led to a significant decline in the incidence of mumps in several countries. MuCV represents a monovalent vaccine but can also be used in combination with other vaccines, such as the measles–mumps–rubella vaccine and multi-valent MuCVs, are commonly used. Occasional outbreaks continue to occur despite the increase in populations vaccinated with MuCV. Previous studies have examined laboratory-confirmed mumps during outbreak periods is regional and from small samples [4]. Thus, it is important to demonstrate adequate effectiveness to provide reassurance regarding the vaccine’s continued capability to confer protective immunity. However, studies clinically confirming mumps have been routinely carried out to measure the effectiveness of MuCV. Moreover, a certain level of latency and misdiagnosis has been observed in the clinical diagnosis of mumps. Most clinical studies consist of serum neutralization tests for mumps antibodies, which can measure the clinical and serologic responses to the MuCV rather than the true effectiveness of the MuCV. Some reports indicate that the diagnosis of mumps can be confirmed in the laboratory by the isolation of the mumps virus from specimens, which may reflect the effectiveness of MuCV [5,6,7].

The most common adverse events related to MuCV include injection-site pain, redness, and swelling; fever, vomiting, and drowsiness; and orchitis and skin infection. Serious adverse events (SAEs) have primarily consisted of skin infections and obstructive bronchitis and inflammatory diseases, which include orchitis, parotitis, meningitis, and pancreatitis [8,9,10]. Prevention of the adverse events associated with MuCV at different doses is becoming an increasingly critical global public health issue as an increased risk of safety events after doses of immunization have been reported [11,12]. Taken together, the present meta-analysis was conducted to synthetically assess the effectiveness and safety of MuCV in preventing laboratory-confirmed mumps cases from 2002–2017.

## 2 Materials and methods

### 2.1 Literature search

We searched PubMed, Embase, and Cochrane Library searchers databases for studies published from inception to May 3, 2019. Additionally, based on the importance of mumps in China, we used the China National Knowledge Infrastructure databases, as well as the theses and dissertations database in China. The inclusion criteria included case–control studies, cohort studies, and randomised control trials (RCTs). Key words or subject headings that were used consisted of “mumps-containing vaccine,” “effectiveness,” and “safety.” We scanned the relevant articles retrieved from the reference lists of the eligible articles. The Preferred Reporting Items for Systematic Reviews and Meta-Analysis protocol was performed in the application of this systematic review and meta-analysis.

### 2.2 Inclusion and exclusion criteria

**Figure 1:**
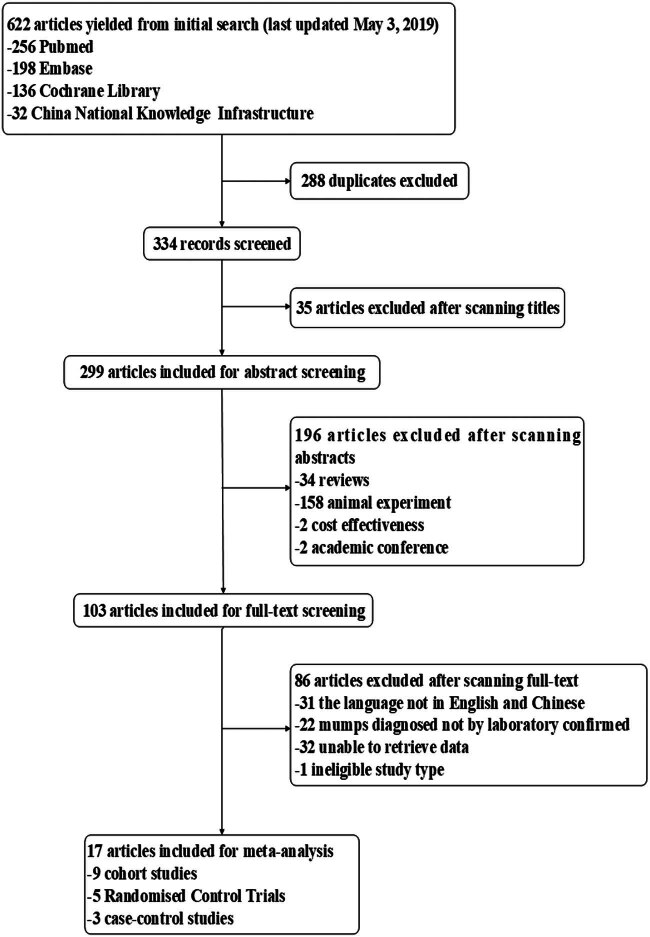
Selection of studies in our meta-analysis: a total of nine cohort studies, five RCTs, and three case–control studies were ultimately included in this meta-analysis.

Studies assessing the effectiveness and safety of MuCV in participants from 2002 to 2017 were included for analysis. Included study types consisted of RCTs, cohort, descriptive, and case–control studies. Studies involving animal trials were excluded. Following the exclusion of 288 duplicates, abstracts and full texts were screened (Figure 1). Finally, the trial and control groups were extracted from the RCTs, the case and control groups from case–control studies, and the exposed and non-exposed groups from cohort studies. Some descriptive studies from which we could not identify two groups were excluded. For the RCTs, the control groups included placebo treatments, such as sodium chloride (4.5 mg), sucrose (12.5 mg), or a combination of these. Studies were excluded if they considered molecular biology, vaccine development, animal studies, and reviews, or were not published in either English or Chinese. Corresponding authors were contacted via email or telephone when critical data were defaulted in the original studies. Single studies associated with duplicate publications were considered one study and the publication with the most complete information was selected.

### 2.3 Outcome assessment

The effectiveness of MuCV after the last dose was calculated with 95% confidence interval (CI) in each study. The secondary outcomes were adverse events. We primarily considered injection pain, redness, and swelling to be local symptoms. Fever, vomiting, and drowsiness were considered systematic reactions. The SAEs primarily consisted of inflammatory diseases, skin infections, and obstructive bronchitis. For a better understanding of the safety issues of MuCV, we compared different doses of MuCV.

### 2.4 Data extraction

All data were extracted by the lead author (LFH), and the second author (GBG) summarized the findings in Excel. Any disagreements in the data were resolved through discussion with the third author (PX). The following characteristics that might influence the MuCV effectiveness and safety were retrieved, including the first author, year of publication, study design, country, study period, intervention, type and valent of MuCV, coverage of MuCV, percentage of males, sample extracted from the participants, effectiveness confirmed index, and information sources. All of the above data were extracted into prepared templates.

### 2.5 Quality assessment

The Cochrane’s collaboration’s tool for assessing the risk of biases was used to evaluate the biases in the RCTs (http://www.cochrane-handbook.org). We assessed seven biases including the selection bias, attribution bias, performance bias, reporting bias, detection bias, and other biases. In addition, the tool was used to assess the quality of the five RCTs. Regarding non-randomized experimental studies, we used the Newcastle–Ottawa Scale (NOS) to evaluate the quality of nine cohort studies and three case–control studies, including the four fields of selection, comparability, outcome, and total score (http://www.ohri.ca/programs/clinical_epidemiology/oxford.asp). We divided the NOS score into three levels: (1) high quality, score ≥7; (2) moderate quality, 4 ≤ score < 7; and (3) low quality, score <4 [13].

### 2.6 Statistical analysis

All statistical calculations were performed using Revman Manager 5.3 and Stata (version 12.0). We calculated the summary relative risk (RR) and odds risk (OR) estimates and 95% CIs as association measures for dichotomous results at the 5% level. Since the OR was used to evaluate the risk of disease in case–control studies, whereas RR was used to evaluate cohort studies and RCTs, we evaluated the risk of laboratory-confirmed mumps from the case–control studies, cohort studies, and RCTs, respectively.

The *I* ^2^ statistic was used to assess the level of heterogeneity across the included studies, with values of 25, 50, and 75%, which represented low, moderate, and high heterogeneities, respectively [14]. We initially performed the analyses using a random-effects model, which were re-adjusted using a fixed-effects model in case the *I* ^*2*^ value was lower than 50% and the *P* value was greater than 0.05. All of the statistical results were displayed as forest plots and tables.

We performed a subgroup analysis to compare the pooled association estimates and heterogeneity. The subgroup analyses were related to outbreak versus non-outbreak period, as well as one dose of MuCV versus two and three doses of MuCV. Since there is only one study that has reported three doses of MuCV making it impossible to compare to other studies, we evaluated the groups that received two and three doses group compared with the one dose group. Potential outliers were detected using a sensitivity analysis by removing each estimate one at a time and recalculating the pooled estimates. We also performed sensitivity analyses by restricting ORs or RRs adjusted for potential confounding factors. Begg’s funnel plots were used to assess publication bias. Since the present study consisted of a meta-analysis and the patients were not directly involved, patient consent was not required. This study was approved by the Ethics Committee of Guangdong Medical University.

## 3 Results

### 3.1 Literature search

The database search identified 622 articles containing relevant studies and clinical trial experiments. The most ineligible studies were excluded based on duplicates and abstracts and titles. Of the 622 selected articles, a total of 288 duplicates were excluded. Of the 334 screened records, 35 articles were excluded after scanning the titles. Of the 299 studies included for abstract screening, 196 articles were excluded after scanning the abstracts. Finally, 17 articles were included in our analysis after excluding 86 articles due to the full text. A total of nine cohort studies, five RCTs, and three case–control studies were ultimately included in this meta-analysis. The details of the selection process details are presented in Figure 1.

### 3.2 Description characteristics

The included studies were conducted in Belgium, Canada, China, Finland, Germany, Italy, Jordan, Korea, Spain, the United Kingdom, the United States, the Netherlands, and Taiwan, China [5,15–30]. The total number of participants ranged from 20 to 3,808,130. The confirmed cases primarily occurred in children aged approximately 13 years old. Various valents and types of MuCV were used. The case definition intended for detecting laboratory-confirmed mumps was performed using polymerase chain reaction (PCR) by the presence of mumps virus RNA in all studies. As supplemental indexes, immunoglobulin G (IgG) antibody was assessed in 15 studies, IgM antibody was assessed in 11 studies from patient samples (e.g., blood, oral fluid, and throat swabs). Nine studies used all the three methods described above. Regionally, the department of Centers for Disease Control and Prevention (CDC) was the most common resource for recording information regarding laboratory-confirmed mumps and a history of MuCV vaccination. The department of CDC included China, the United States, and Korea. The detailed characteristics are listed in Table 1.

**Table 1:** Characteristics of included studies in our meta-analysis

Author (year) [ref]	Country	Study period	Intervention	Mumps-containing vaccine		Percent male	Sample extracted from participants	Laboratory confirmed index	Information sources
				Valent	Type				
**5 RCTs**									
Yan (2014) [25]	China	October 2012 to November 2012	NA	NA	SP-A viral of the F-genotype	43.00%	Throat swabs, blood	RNA, IgG	Chinese Provincial Center for Disease Control and Prevention
Vesikari (2010) [23]	Finland	October 2006 to March 2007	Subcutaneous injection in deltoid region	Quadruvalent	Jeryl Lynn	48.90%	Blood	RNA, IgG	NA
Deichmann (2015) [29]	Germany	NA	Muscle injection in deltoid region	Quadruvalent and hexavalent	Jeryl Lynn	NA	Blood	RNA, IgG, IgM	NA
Huang (2014) [27]	Taiwan	August 2010 to July 2012	NA	Trivalent	Jeryl Lynn	51.11%	Blood	RNA, IgG, IgM	University laboratory
Klein et al. (2012) [20]	The United States	NA	Subcutaneous injection of left thigh	Quadruvalent	Jeryl Lynn	50.62%	Blood	RNA, IgG, IgM	NA
**9 cohort studies**									
Deeks et al. (2011) [15]	Canada	September 2009 to June 10 2010	NA	Trivalent	NA	72.40%	NA	RNA, IgM	The Ontario Immunization Record Information System database
Batayneh (2002) [22]	Jordan	1999 to 2000	NA	Trivalent	NA	51.28%	Blood	RNA, IgG	The Department of Notifiable Communicable Diseases
Braeye (2014) [21]	Belgium	June 2012 to April 2013	NA	Trivalent	Jeryl Lynn	53.00%	NA	RNA, IgG, IgM	The National Research Center
Ogbuanu (2012) [31]	The United States	January to February, 2010	NA	Trivalent	Jeryl Lynn	43.99%	NA	RNA, IgG, IgM	The United States Center for Disease Control and Prevention and the New York State Department of Health.
La (2017) [28]	Italy	2009 to 2011	NA	Quadruvalent	NA	50.30%	NA	RNA, IgG, IgM	Vaccination register and hospital discharge records
Schaffzin et al. (2007) [19]	The United States	June to September, 2005	NA	Trivalent	Jeryl Lynn et al.	47.00%	Blood	RNA, IgG, IgM	The United States Center for Disease Control and Prevention
Dittrich (2011) [16]	The Netherlands	2003 to 2006	NA	Trivalent	Jeryl Lynn	51.34%	Oral fluid	RNA, IgG	The vaccination register
Kawano et al. (2015) [17]	The United States	September 2005 to December 2013	NA	Monovalent	Hoshino strain	62.50%	Blood and oral fluid	RNA, IgG	Laboratory medical records
Guo (2016) [30]	China	NA	NA	Trivalent	NA	45.71%	Blood	RNA, IgG	NA
**3 case**–**control studies**									
Castilla (2009) [17]	Spain	August 2006 to June 2008	NA	Trivalent	Jeryl Lynn	48.00%	NA	RNA, IgG, IgM	The regional vaccination registry
Moon (2017) [26]	Korea	2012	NA	Trivalent	Jeryl Lynn or RIT 4385 strains	100%	Blood	RNA, IgG, IgM	The Korea Center for Disease Control and Prevention
Harling (2005) [25]	The United Kingdom	June 1998 to May 1999	NA	Trivalent	NA	37.61%	Oral fluid	RNA, IgM	The local Consultant in Communicable Disease Control

*Note.* Ref: reference; SD: Standard difference; RCTs: Ran.

### 3.3 Vaccine effectiveness (VE) against laboratory-confirmed mumps

#### 3.3.1 Effectiveness in case–control studies

**Figure 2:**
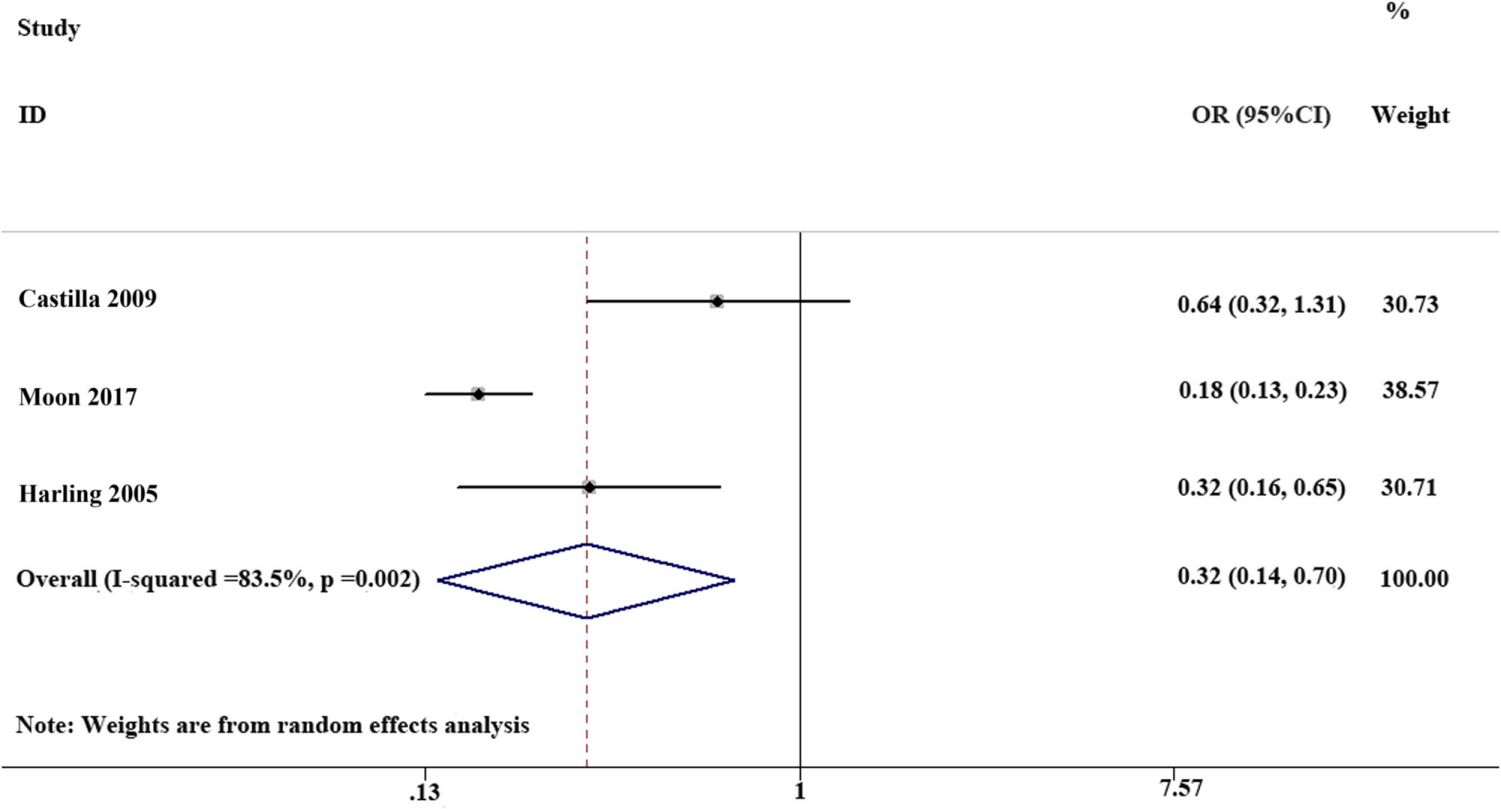
The effectiveness of mumps containing vaccine (3 case–control studies): of the three case–control studies, the VE of MuCV in preventing laboratory-confirmed mumps was 68%.

Of the three case–control studies, the VE of MuCV in preventing laboratory-confirmed mumps was 68% (OR: 0.32; 95% CI, 0.14−0.70). In total, of the 1,140 individuals in the laboratory-confirmed mumps group, 130 were MuCV vaccinated, and of the 1,258 individuals in the control group, 397 were vaccinated. The above comparison was conducted using a random-effects model (Figure 2).

#### 3.3.2 Effectiveness in cohort studies and RCTs

Of the nine cohort studies and five RCTs, we compared the mumps incidence rate between the vaccination group and the control group. The results showed that the overall VE for preventing laboratory-confirmed mumps was 58% (RR: 0.42; 95% CI, 0.26−0.69).

#### 3.3.3 Effectiveness between the outbreak and non-outbreak period

**Figure 3:**
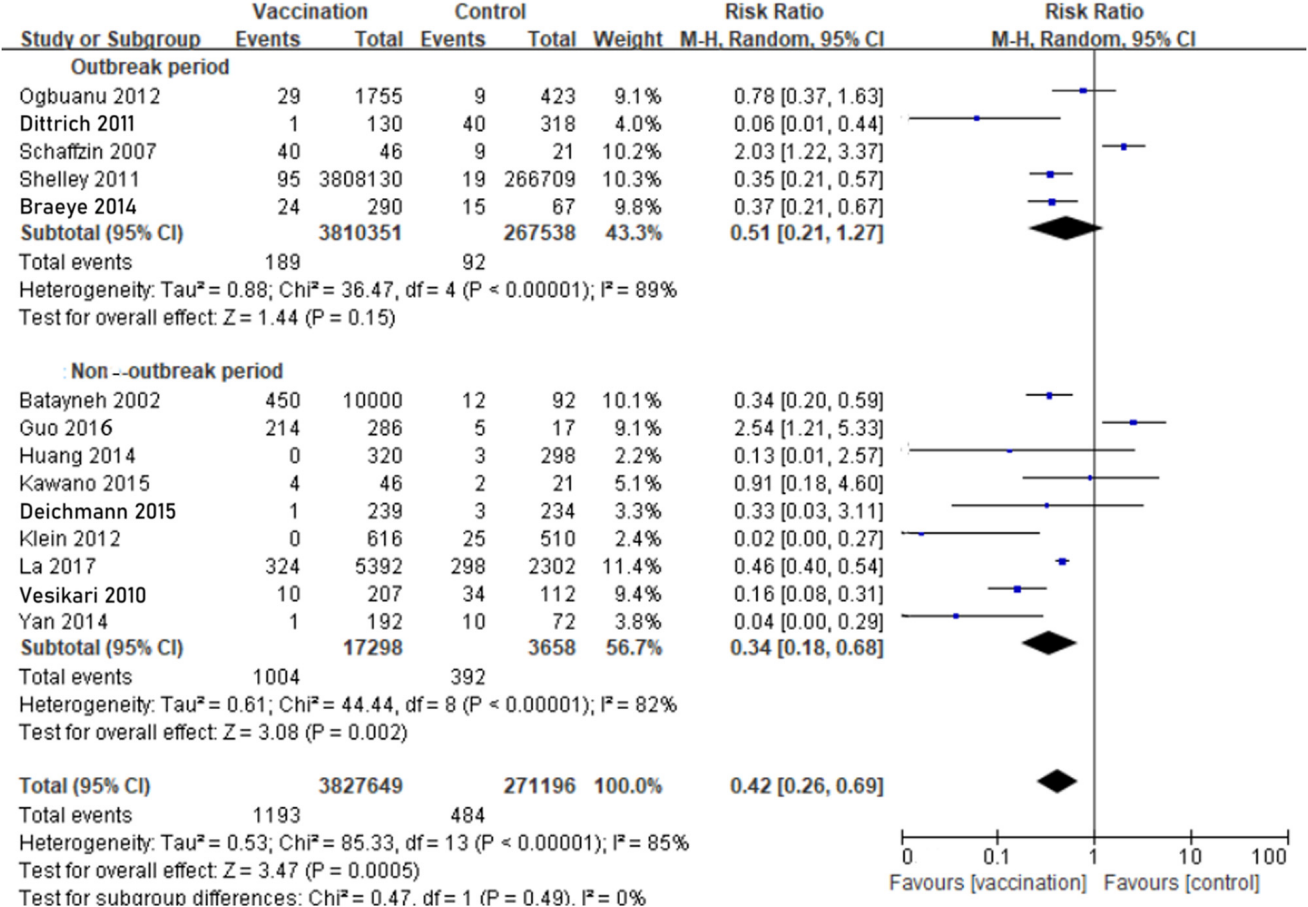
Comparison between outbreak and non-outbreak period evaluating the effectiveness of mumps containing vaccine (9 cohort studies and 5 RCTs): during the outbreak period, the VE of MuCV was 49% (RR: 0.51; 95% CI, 0.21−1.27), whereas the VE during the non-outbreak period was 66% (RR: 0.34; 95% CI, 0.18−0.68).

During the outbreak period, we estimated the incidence was 0.50‱ in the MuCV vaccination group, which was calculated from 189 people diagnosed with laboratory-confirmed mumps among the 3,810,351 MuCV recipients. Moreover, the incidence was 3.44‱ in the control group, which was calculated from 92 people diagnosed with laboratory-confirmed mumps among the 267,538 non-MuCV recipients. The outbreak and non-outbreak periods were used as comparisons. During the outbreak period, the VE of MuCV was 49% (RR: 0.51; 95% CI, 0.21−1.27), whereas the VE during the non-outbreak period was 66% (RR: 0.34; 95% CI, 0.18−0.68). Although the pooled RRs showed that VE of MuCV during non-outbreak was superior than the outbreak period, the 95% CI was similar among the outbreak group and the non-outbreak group, which indicates that VE of MuCV during outbreak was no better than non-outbreak period. All three of the above comparisons were applied in a random-effects model (Figure 3).

#### 3.3.4 Effectiveness in one, two, and three doses

**Figure 4:**
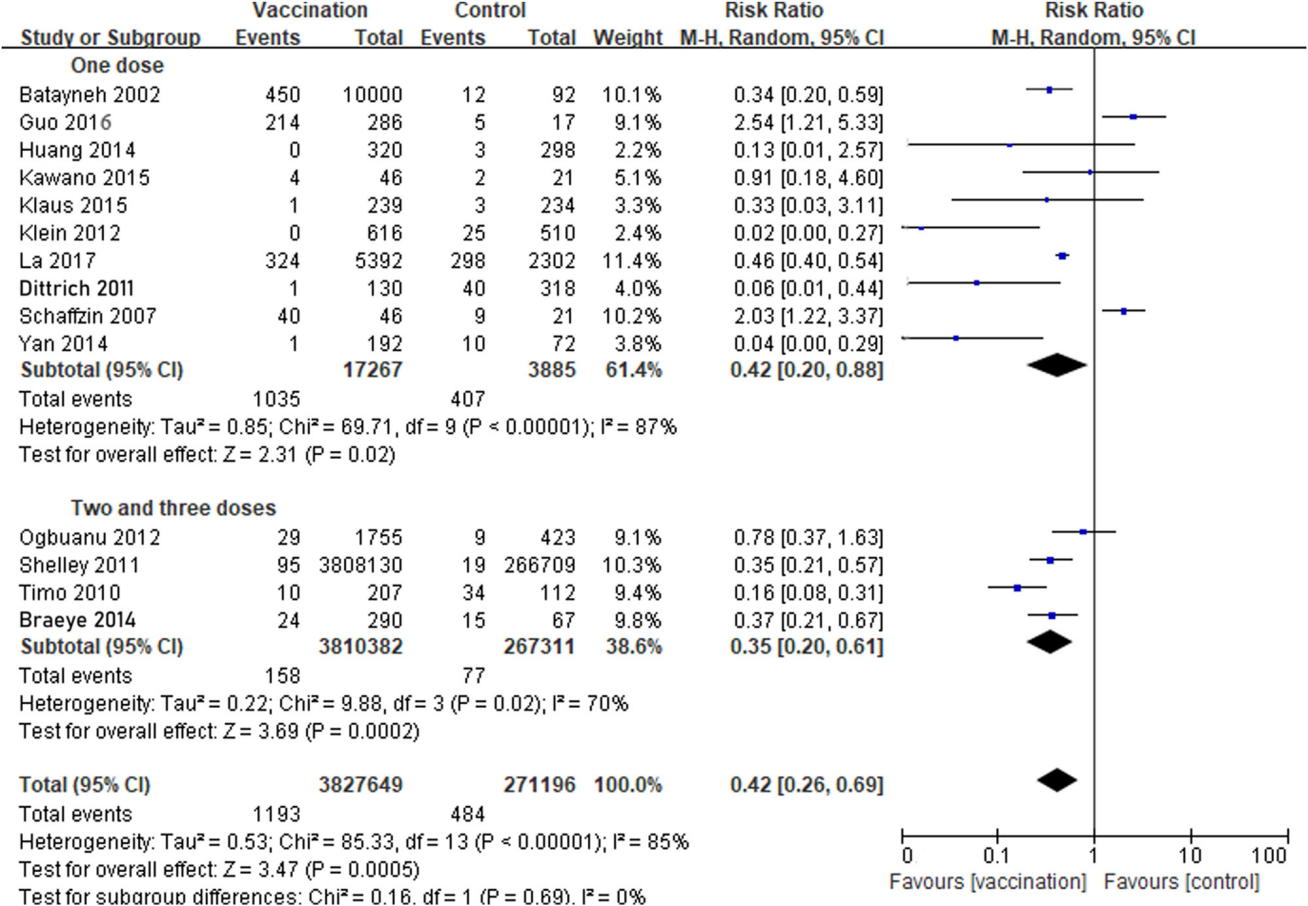
Comparison between one dose, two, and three doses evaluating the effectiveness of mumps containing vaccine (9 cohort studies and 5 RCTs): the results showed that the VE of one dose of MuCV was 58% (RR 0.42; 95% CI, 0.20−0.88), whereas two and three doses were 65% (RR 0.35; 95% CI, 0.20−0.61).

There were ten studies that reported one dose of MuCV, three studies that reported two doses, and one study that reported three doses. The results showed that the VE of one dose of MuCV was 58% (RR 0.42; 95% CI, 0.20−0.88), whereas two and three doses were 65% (RR 0.35; 95% CI, 0.20−0.61). However, the 95% CIs of the two subgroups showed an overlap, which indicated that there was no statistically significant difference between the two subgroups. All the above comparisons were applied in a random-effects model (Figure 4).

### 3.4 Safety

To assess safety, five RCTs and one cohort study added up to six studies that investigated adverse events. In most of the studies, local symptoms, systematic symptoms, or SAEs occurred during the first 15 days of the post-vaccination observation period. Redness and fever were the two most common symptoms. The incidence rate of two local symptoms and two systematic symptoms following two doses of MuCV was higher than that experienced following one dose, which was statistically different.

#### 3.4.1 Local symptoms

The five RCTs and one cohort study reported 701 cases among 1,574 participants in the MuCV group and 248 cases among the 1,262 participants in the control group. All three symptoms occurred until 56 days post-vaccination. For injection-site pain, 25.71% of events associated with one dose of the MuCV were reported, which were higher than that of two doses. In addition, we found that two doses of MuCV statistically increased the risk of swelling and redness. There were insufficient data available to evaluate the other symptoms which were not depicted in Table S1.

#### 3.4.2 Systematic symptoms

The number of systematic symptoms was 877 cases among the 1,335 participants in the MuCV group and 461 cases among the 1,028 participants in the control group. Statistical differences existed regarding fever (RR: 1.68; 95% CI, 1.36−2.08; *I* ^*2*^ = 0%) and drowsiness (RR: 1.63; 95% CI, 1.34−1.98; *I* ^*2*^ = 43%). Together with vomiting, all three of these symptoms showed that the incidence rate associated with two doses of MuCV were higher, although vomiting was not statistically significant.

#### 3.4.3 SAEs

Four cases (0.97%) of skin infection and obstructive occurred after two doses of the MuCV vaccination. No cases of orchitis existed and there are no related fatal SAEs reported in any of the included studies. Nevertheless, the two symptoms were not statistically significant.

### 3.5 Quality and risk of bias assessment

Taken together, a total of eleven studies were of moderate quality and the other six studies were of high quality. In considering the five RCTs, the overall quality was good and indicated that the overall risk of bias was low (Figure S1). Except for detection and performance, bias primarily emerged as high risk of biases, the other five biases mostly emerged as low risk of biases. With regard to the nine cohort studies and three case–control studies, six studies were assigned as moderate quality (score of 6 for four studies and score of 5 for two studies) and the other six studies were assigned as high quality, as shown in Table S2 according to the NOS. The sensitivity analysis confirmed the credibility of this meta-analysis (Figure S2). The funnel plot displayed non-significant asymmetry (Begg’s test *P* > 0.05) both in the three case–control studies, nine cohort studies, and five RCTs (Figure S3).

## 4 Discussion

This is the first systematic review and meta-analysis that has evaluated the effectiveness of MuCV using laboratory diagnosis and reassessed the safety of MuCV. To our knowledge, the reasons for the increasing instances of mumps outbreaks are multifaceted. One significant possibility may be the decreasing effectiveness of the MuCV. Laboratory confirmation has been found to be of high value to diagnose mumps and decrease the possibility that laboratory confirmation could be the reason for the onset of mumps outbreaks [5,15–30]. Thus, evaluating the effectiveness and safety of MuCV for decreasing laboratory-confirmed mumps is of high value and there are an insufficient number of previously studied regional samples [4]. Our findings provide a reference for countries recording information pertaining to laboratory confirmation and may reflect a need to re-evaluate the precise VE and safety of MuCV.

In general, there are insufficient data regarding whether MuCV is effective for laboratory-confirmed mumps and related concerns continue to increase. Instead of laboratory-confirmed mumps, a large body of evidence has been derived from cases of clinical mumps [32]. Therefore, biases and residual confounding factors provide alternative explanations for the incorrect VE of MuCV. The results of this study show that the effectiveness of MuCV in cohort studies and RCTs was lower for laboratory-confirmed mumps (VEs ranging from 49 to 66% with a pooled estimates of 58%) compared to approximately 66.4% after an individual’s final vaccine dose as reported as clinical-confirmed cases by previous analyses [33,34]. A possible explanation for this is that the studies included in our meta-analysis focused on laboratory-confirmed cases rather than clinical-confirmed cases. As a result, a greater number of mumps cases were found than previously reported studies, most of which focused on clinically diagnosed mumps. Sabine et al. indicated that for every one case of clinical vaccinated mumps, at least three asymptomatic cases can be expected and at higher risk of mumps virus infection [16]. On the other hand, a significant decrease in the voluntarily MuCV-vaccinated population explains the reduction of VEs to some extent [35]. Those with immunity against mumps (i.e., those with laboratory-confirmed cases) reflect the true level of herd immunity [36]. In addition, the proportion of the overall VE was lower than the suggested threshold of 90−92% to interrupt transmission [35].

In reducing the cases of laboratory-confirmed mumps, the results showed that MuCV during the non-outbreak period was more effective than that during the outbreak period (VE = 49% during the outbreak period versus 66% during the non-outbreak period). However, the 95% CIs indicate similar VEs between the two periods, which differed from that of previous studies. To our knowledge, the outbreaks were usually associated with higher MuCV effectiveness and the degree of protection increased at the beginning of 20th century [37]. The similarity of the VE intervals illustrated a decreased frequency of protection in the population exposed to mumps virus during outbreak period. The key contributing factor to this phenomenon has been proposed to be waning immunity, which was potentially due to immune escape resulting from various valents and types of MuCV over a long period. Waning immunity indicated a waning of vaccine-induced immunity, which increased the susceptibility to infection with the mumps virus since a mismatch existed between the vaccine strain and a predominant circulating wild-type strain of the mumps virus [38,39]. In addition, cases of reinfection have been reported among individuals who have had naturally acquired mumps, suggesting that MuCV may not be effective in some individuals and VE of MuCV might be overvalued [38]. From this perspective, waning immunity would exert an influence on decreasing the robustness of vaccine-derived immunity and exponentially increase the probability of mumps virus exposure during the outbreak period. In fact, most mumps cases in recent outbreaks have been reported among young adults, and some researchers estimated that may be due to waning immunity towards MuCV [40,41].

Compared with only one dose of the vaccine in 17,267 individuals, the participants vaccinated with two doses reached 3,808,627 resulting from an immunization with two doses of MuCV as part of a childhood vaccination program in many vaccinated countries by 2005 [40,41]. Consistent with most studies, the point estimate of the pooled VE for two and three doses (VE = 65%) was higher than that of one dose (VE = 58%). Specifically, the pooled analysis reported an impressive remission rate of VEs of MuCV in laboratory-confirmed mumps. Effectiveness of one dose of MuCV was shown from 66 to 96%, whereas the effectiveness of two doses and three does was observed from 86 to 99% according to previous studies [42–45]. However, the 95% CI of the two subgroups indicated that the effectiveness did not differ significantly between two or three doses of MuCV and one dose of MuCV. One possible explanation for this finding may be that many individuals who reported being vaccinated with one dose may have been vaccinated with another dose and boosted by exposure via an increasing occurrence of outbreaks, but their history of vaccination has been incorrectly recorded [31]. In addition, Deeks et al. found that when vaccine supplies were limited, administering a single dose of the vaccine may avert more cases and deaths than a standard two-dose campaign [15]. Furthermore, there were only three studies involving a two doses MuCV vaccine regimen. Widespread third-dose intervention of MuCV can be extremely time consuming and resource intensive; thus, we could extract only one study involving a three-dose MuCV study design [32,46]. To date, there are fewer studies that investigated two and three doses of MuCV compared to that involving one dose of MuCV [43]. Thus, our results may be associated with limitations due to the lack of studies on two and three doses of MuCV. In general, MuCV was found to be effective for laboratory-confirmed mumps and which doses of MuCV should be recommended as being in need of further research.

Some confounding factors that were identified may explain our insignificant findings. For the detection of laboratory-confirmed mumps, three methods were used. Qualitative RNA expression by PCR was a determinant factor, while the levels of IgG and IgM were considered supplemental factors. While some studies used all three methods, others used only one of the three. Although PCR was found to be significant for identifying mumps virus infection, the data were not directly provided. Therefore, we either contacted the authors or indirectly obtained the data through calculations. Thus, our task was a difficult and time-consuming process. The evaluation of VE by cautiously confirming PCR test results may provide better understanding of the precise VE of the MuCV. There have been no relevant placebo RCTs included in this analysis which is another possible confounding factor. It is considered unethical to withhold MuCV, particularly from those at the highest risk of developing severe illness due to mumps.

Safety issues concerning post-vaccination symptoms associated with different doses of MuCV are a concern [47]. In addition to injection-site pain, the incidence of two local symptoms and three systematic symptoms increased with additional doses of MuCV. Redness, swelling, and drowsiness were the most frequently reported symptoms common among the majority of the studies [10,12]. Compared with previous studies, the higher reporting of adverse events may be due to the increasing number of MuCV-vaccinated individuals or the availability of a variety of new MuCV [48,49]. Rare SAEs and the lack of mortality related to MuCV indicate that MuCV was generally and unequivocally safe for laboratory-confirmed mumps in line with what has been reported globally.

With respect to the limitations associated with our analysis, including moderate to high heterogeneity, the present results should be interpreted with caution. First, there have been few studies conducted on MuCV vaccination and evaluated by laboratory confirmation. In some studies, the data were limited, making data extraction difficult. Thus, we were required to contact the corresponding authors. Moreover, some of the studies included in this analysis were observational studies and could have led to detection bias.

## 5 Conclusion

Our results suggest that MuCV provides lower protection against laboratory-confirmed mumps. MuCV showed similar effectiveness during an outbreak period and a non-outbreak period. Moreover, MuCV did not show increased effectiveness with the delivery of two and three doses rather than one dose. MuCV exhibited acceptable adverse event profiles. The confirmation of laboratory-confirmed mumps will help to more precisely assess the VE and safety of MuCV.

## Supplementary Material

Supplementary material
