# Supplementary material for: Effectiveness and safety of a mumps containing vaccine in preventing laboratory-confirmed mumps cases from 2002 to 2017: A meta-analysis
Source: Open Life Sci. 2024 Feb 8;19(1):20220820. doi: 10.1515/biol-2022-0820 (PMC10921504; doi:10.1515/biol-2022-0820)
Supplement: Supplementary material [file biol-2022-0820-sm.pdf]

Supplementary material

Figures S1–S3 and Tables S1 and S2

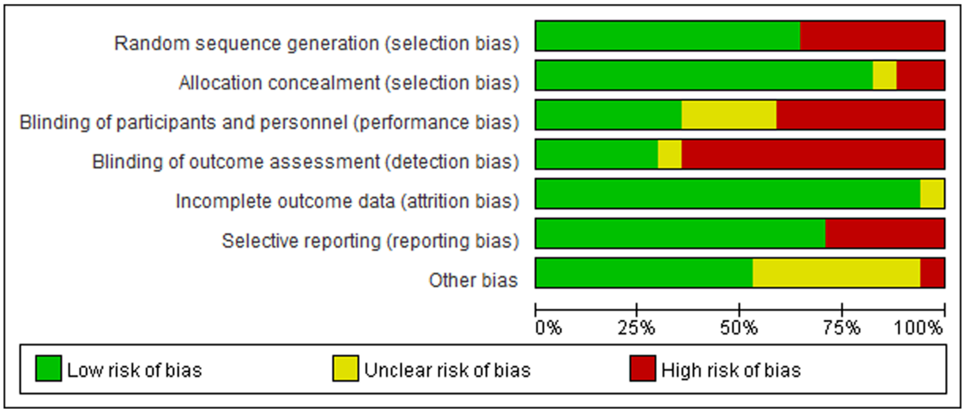

Figure S1: The risk of bias summary of 5 RCTs by the Cochrane's collaboration's tool for assessment risk of bias: a total of eleven studies were of moderate quality and the other six studies were of high quality.

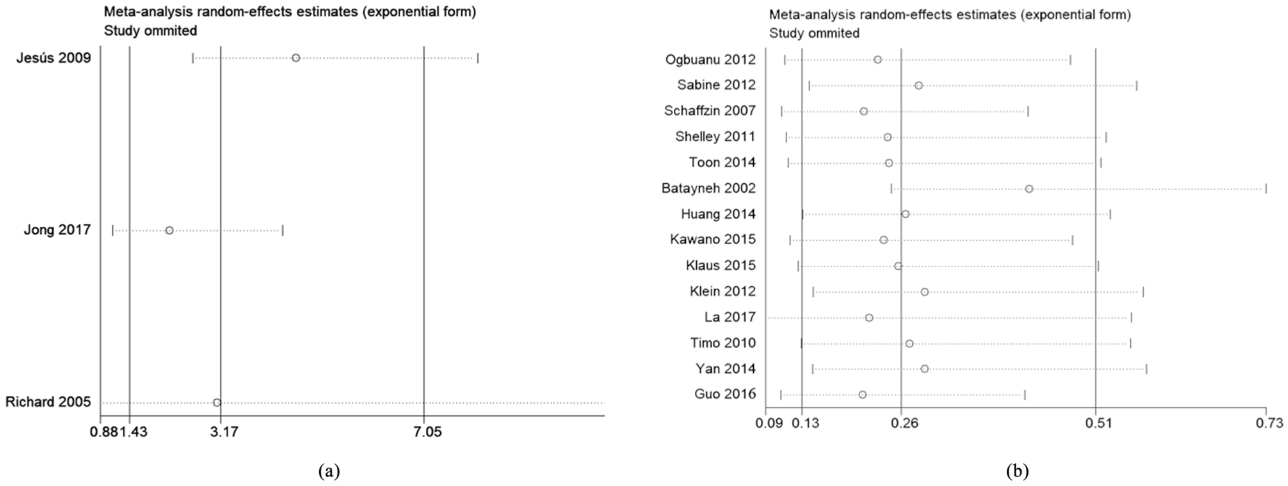

Figure S2: Sensitivity analysis of studies in our meta-analysis.

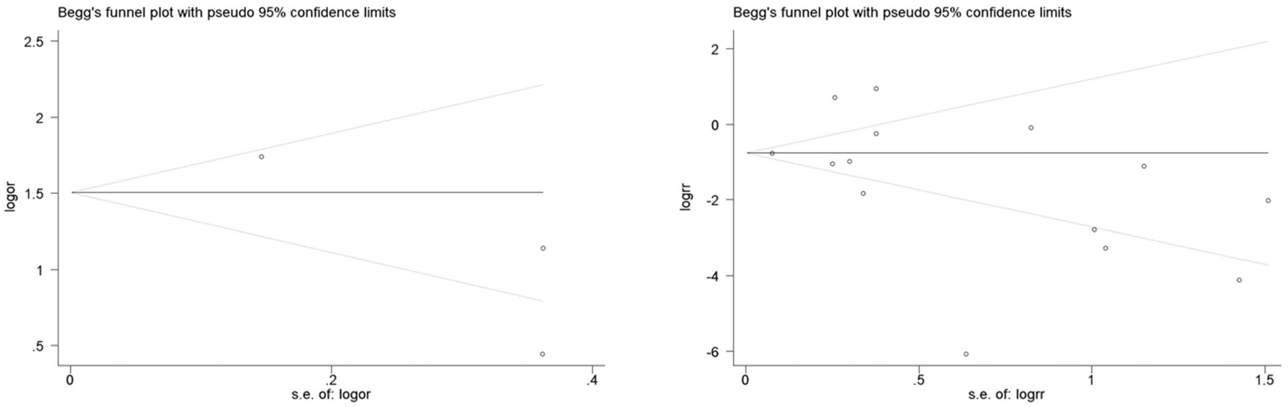

A B

Figure S3: Publication bias of studies in our meta-analysis: the funnel plot displayed non-significant asymmetry (Begg's test $P > 0.05$) both in the three case-control studies, nine cohort studies, and five RCTs.

Table S1: Safety events of mumps-containing vaccine in different doses assessed by RR index (5 RCTs and 1 cohort study)

Reaction or events	Instance of one dose (%)	Instance of two doses (%)	No. of studies	RR (95% CI)	Test heterogeneity (I^2)	*P* value
**Local symptoms**						
Injection-site pain	25.71	20.50	4	5.16 (2.97–8.95)	15%	< 0.001
Redness	7.64	44.95	4	1.86 (1.51–2.29)	47%	< 0.001
Swelling	2.41	20.86	5	5.19 (3.14–8.27)	90%	0.008
**Systematic symptoms**						
Fever	10.89	47.83	6	1.68 (1.36–2.08)	0%	0.003
Vomiting	7.68	21.76	4	1.19 (0.84–1.27)	49%	0.33
Drowsiness	22.52	28.52	6	1.63 (1.34–1.98)	43%	<0.001
**Serious Adverse Events**						
Orchitis	0	0	6	2.06(0.21–19.85)	0%	0.53
Skin infection and obstructive	0	0.97	6	3.44 (0.40–29.57)	0%	0.26

RR: Relative Risk; No: Numero; CI: Confidence Interval.

Table S2: NOS score of cohort studies and case-control studies included in our meta-analysis

Author (year) [ref]	Selection	Comparability	Outcome	Total score
**9 cohort studies**				
Deeks (2011) [1]	3	2	1	6
Batayneh (2002) [7]	2	2	2	6
Toon (2014) [6]	4	2	2	8
Ogbuanu (2012) [12]	4	2	1	7
La (2017) [10]	2	2	2	6
Schaffzin (2007) [5]	3	2	1	6
Sabine (2012) [2]	3	1	3	7
Kawano (2015) [3]	4	2	2	8
Guo (2016) [11]	2	2	1	5
**3 case-control studies**				
Jesús (2009) [4]	3	2	2	7
Jong (2017) [9]	2	1	2	5
Richard (2005) [8]	4	2	2	8

NOS: The Newcastle-Ottawa Scale; Ref: Reference.

References

[1] Deeks SL, Lim GH, Simpson MA, Gagné L, Gubbay J, Kristjanson E, et al. An assessment of mumps vaccine effectiveness by dose during an outbreak in Canada. CMAJ. 2011;183(9):1014–20.

[2] Dittrich S, Hahné S, van Lier A, Kohl R, Boot H, Koopmans M, et al. Assessment of serological evidence for mumps virus infection in vaccinated children. Vaccine. 2011;29(49):9271–5.

[3] Kawano Y, Suzuki M, Kawada J, Kimura H, Kamei H, Ohnishi Y, et al. Effectiveness and safety of immunization with live-attenuated and inactivated vaccines for pediatric liver transplantation recipients. Vaccine. 2015;33(12):1440–5.

[4] Castilla J, García Cenoz M, Arriazu M, Fernández-Alonso M, Martínez-Artola V, Etxeberria J, et al. Effectiveness of Jeryl Lynn-containing vaccine in Spanish children. Vaccine. 2009;27(15):2089–93.

[5] Schaffzin JK, Pollock L, Schulte C, Henry K, Dayan G, Blog D, et al. Effectiveness of previous mumps vaccination during a summer camp outbreak. Pediatrics. 2007;120(4):e862–8.

[6] Batayneh N, Bdour S. Mumps: immune status of adults and epidemiology as a necessary background for choice of vaccination strategy in Jordan. Apmis. 2002;110(7–8):528–34.

[7] Vesikari T, Karvonen A, Lindblad N, Korhonen T, Lommel P, Willems P, et al. Safety and immunogenicity of a booster dose of the 10-valent pneumococcal nontypeable Haemophilus influenzae protein D conjugate vaccine coadministered with measles-mumps-rubella-varicella vaccine in children aged 12 to 16 months. Pediatr Infect Dis J. 2010;29(6):e47–56.

[8] Moon JY, Jung J, Huh K. Universal measles-mumps-rubella vaccination to new recruits and the incidence of mumps in the military. Vaccine. 2017;35(32):3913–6.

[9] Huang LM, Lin TY, Chiu CH, Chiu NC, Chen PY, Yeh SJ, et al. Concomitant administration of live attenuated Japanese encephalitis chimeric virus vaccine (JE-CV) and measles, mumps, rubella (MMR) vaccine: Randomized study in toddlers in Taiwan. Vaccine. 2014;32(41):5363–9.

[10] Deichmann KA, Ferrera G, Tran C, Thomas S, Eymin C, Baudin M. Immunogenicity and safety of a combined measles, mumps, rubella and varicella live vaccine (ProQuad®) administered concomitantly with a booster dose of a hexavalent vaccine in 12–23-month-old infants. Vaccine. 2015;33(20):2379–86.

[11] Maillet M, Bouvat E, Robert N, Baccard-Longère M, Morel-Baccard C, Morand P, et al. Mumps outbreak and laboratory diagnosis. J Clin Virol. 2015;62:14–9.

[12] Maglione MA, Das L, Raaen L, Smith A, Chari R, Newberry S, et al. Safety of vaccines used for routine immunization of U.S. children: a systematic review. Pediatrics. 2014;134(2):325–37.
